# Nucleoporin's Like Charge Regions Are Major Regulators of FG Coverage and Dynamics Inside the Nuclear Pore Complex

**DOI:** 10.1371/journal.pone.0143745

**Published:** 2015-12-11

**Authors:** Mohaddeseh Peyro, Mohammad Soheilypour, Ali Ghavami, Mohammad R. K. Mofrad

**Affiliations:** Molecular Cell Biomechanics Laboratory, Departments of Bioengineering and Mechanical Engineering, University of California, Berkeley, California, United States of America; University of Toronto, CANADA

## Abstract

Nucleocytoplasmic transport has been the subject of a large body of research in the past few decades. Recently, the focus of investigations in this field has shifted from studies of the overall function of the nuclear pore complex (NPC) to the examination of the role of different domains of phenylalanine-glycine nucleoporin (FG Nup) sequences on the NPC function. In our recent bioinformatics study, we showed that FG Nups have some evolutionarily conserved sequence-based features that might govern their physical behavior inside the NPC. We proposed the ‘like charge regions’ (LCRs), sequences of charged residues with only one type of charge, as one of the features that play a significant role in the formation of FG network inside the central channel. In this study, we further explore the role of LCRs in the distribution of FG Nups, using a recently developed coarse-grained molecular dynamics model. Our results demonstrate how LCRs affect the formation of two transport pathways. While some FG Nups locate their FG network at the center of the NPC forming a homogeneous meshwork of FG repeats, other FG Nups cover the space adjacent to the NPC wall. LCRs in the former group, i.e. FG Nups that form an FG domain at the center, tend to regulate the size of the highly dense, doughnut-shaped FG meshwork and leave a small low FG density area at the center of the pore for passive diffusion. On the other hand, LCRs in the latter group of FG Nups enable them to maximize their interactions and cover a larger space inside the NPC to increase its capability to transport numerous cargos at the same time. Finally, a new viewpoint is proposed that reconciles different models for the nuclear pore selective barrier function.

## Introduction

The Nuclear pore complex (NPC) is the sole gateway for bidirectional macromolecular transport between the cytoplasm and the nucleus [1–4]. Selective and fast translocation of cargos are specific characteristics of this molecular machinery [1]. The NPC features an eightfold radial symmetry, conserved across all eukaryotic organisms [5,6]. It is made up of ~30 different proteins named nucleoporins (Nups) [7,8], around 30% of which are disordered proteins, highly rich in phenylalanine-glycine (FG) repeats [9,10]. These proteins are known to be responsible for the active translocation of cargos through the NPC [1,11–13]. While small molecules and ions (diameters ~5–9 nm) pass through the NPC via passive diffusion, larger cargos (diameters up to 40 nm) can only be transported through an active mechanism. Active transport of cargos is facilitated by a family of proteins called transporters. Specific cargos, possessing nuclear localization signal (NLS), bind to transporters, most importantly karyopherin β family (hereinafter called Kaps), and form a cargo complex to be able to pass the NPC barrier [1,14–18].

Despite extensive research on the underlying mechanisms of the NPC function, there is still no consensus on how cargos are transported through the NPC. So far, a few models have been proposed for nucleocytoplasmic transport. The selective phase model [12,19,20] suggests that FG Nups inside the NPC form a homogeneous meshwork (hydrogel) via the weak interaction between FG motifs. Due to the high affinity of transporters to FG-repeats, active transport of cargo complexes happens through transient breaking of the FG-FG cross-links and melting of the gel by transporters. From another point of view, the virtual gate model [21] introduces the brush-like structure formed by FG Nups inside the NPC as an entropic barrier. This FG Nup brush repels nonspecific cargos, i.e. cargos that have not recruited transporters, while cargo-complexes can overcome this barrier due to their weak, transient interactions with FG-repeats. The reversible collapse model [22] justifies the active transport of cargos through the NPC by a conformational change of FG Nups (toward their anchoring point) in the presence of Kaps. The reduction-of-dimensionality model [23] suggests that the transport of cargos through the NPC is facilitated by a two-dimensional random walk of cargo-complexes over a so-called FG-repeat surface formed on the wall of the transport channel. Lastly, the forest model [24–26] considers various biophysical characteristics of FG Nups, including Stokes radius and dimension of individual domains, and suggests that FG Nups show two distinct categories of disordered domains: FG domain and stalk domain. While stalk domains possess a relaxed polymer conformation, FG domains show a more collapsed coil conformation due to the interactions between their residues. These two distinct regions cooperate to form a forest-like landscape inside the NPC and provide two separate pathways.

Although nucleocytoplasmic transport has been the focus of many computational and experimental studies in the past few decades [11,19,24,27–33], the role of sequence composition of FG Nups has only recently drawn attention amongst researchers of the field [24–26,28,29,34]. It has been observed that compositional changes in the sequences of FG Nups significantly influence the distribution of FG Nups inside the pore [28,29]. In addition to compositional bias of FG Nups, some specific patterns have recently been discovered in their sequences[25,26,34], which are believed to play a major role in regulating the distribution of FG Nups inside the pore. These observations suggest that nucleocytoplasmic transport is strongly dependent upon unique features hidden in the sequences of comprising FG Nups. In line with that, several diseases including primary biliary cirrhosis, cancer, viral infection, triple A syndrome, and Alzheimer have been directly associated to mutations in sequences of different FG Nups [35–37]. Therefore, exploring how these sequence-based features determine the NPC function can significantly improve the current understanding of nucleocytoplasmic transport and its relation to different diseases.

In our recent bioinformatics study[34], we analyzed more than a thousand sequences of FG Nups across different species and identified evolutionarily conserved specific features within their sequences. One of these features is a region of sequence of FG Nups, in which the charged residues are only either positively or negatively charged. We named these regions as “like charge regions” (LCRs). Our results suggested that largest positively charged LCRs are potentially significant regulators of the FG network inside the central channel of the NPC. We focused our study on largest positively charged LCRs and for the sake of brevity will henceforth call them LCRs. We observed that LCRs co-localize with regions in the sequence that feature a high density of FG repeats and polar residues.

Here, we seek to further explore the significance of these evolutionarily conserved LCRs by examining the biophysical behavior of FG Nups, with the ultimate goal of shedding light on the mechanism of nucleocytoplasmic transport and the role of FG Nups in this selective process. We study the role of LCRs in FG network formation via a recently developed coarse-grained molecular dynamics model of the NPC [28]. The role of LCRs will be explored in three different levels: individual FG Nups, rings of FG Nups including one type of FG Nup, and the whole NPC. In the individual FG Nup simulations we explore the effect of LCRs on the dynamics of isolated FG Nups, whereas simulations featuring the FG Nups rings are performed to understand how Nups within each layer of the NPC interact and form FG networks. In a higher scale, simulations involving the entire NPC model would provide further insight on how the interactions of FG Nups within each layer and across different layers promote FG network formation and facilitate transport. At each scale, the conformational physics presented by the wildtype sequences are compared to that in the mutated sequences with charged-to-alanine scanning in the LCR.

## Materials and Methods

A one-bead-per-amino acid coarse-grained molecular dynamics model was used to model the NPC and explore FG network formation [28]. The model is specifically designed and optimized to study biophysical behavior of intrinsically disordered proteins. Amino acids are represented by single beads with a fixed mass and distance of 120Da and 0.38nm, respectively. In all of the simulations, only disordered domains of FG Nups were considered, which are obtained from [24]. The force-field developed for this model accounts for different biophysical factors including bending and torsion potentials between neighboring beads, an implicit solvent, ion screening effect, and hydrophobic and electrostatic interactions. The interaction between FG Nups is governed by a modified Lennard-Jones potential. The Lennard-Jones potential consists of two parts, the first part takes care of the excluded volume interaction while the second part is related to the hydrophobicity, which changes according to the nature of the amino acids. The model has proven accurate and useful in simulating biopolymeric behavior of intrinsically disordered proteins, specifically FG Nups [28,38].

FG Nups were studied in three different levels. First set of simulations was conducted on individual FG Nups. Each Nup was simulated three times, each for 100ns. Second set of simulations was performed on eightfold ring arrangements of FG Nups. Ring arrangement of FG Nups has been used before for studying nucleocytoplasmic transport in sections of the NPC [25,33]. Each simulation contains eight copies of a single type of FG Nup arranged in a ring configuration to represent various regions of the NPC [25]. Diameter of each ring is equal to the diameter of the NPC at the corresponding tethering point of the FG Nup. Rings are 10nm in height, representing the maximum vertical space that each FG Nup sweeps from *in silico* experiments [28]. Each ring was simulated three times, each for 1 μs. Third set of simulations was done on the whole NPC model. The geometrical model used in [28] was utilized, which is based on the geometry of the yeast NPC scaffold and FG Nup anchoring points [31,39]. The scaffold is modeled as a solid surface with only excluded volume interaction with FG Nups. FG Nups are fixed at their corresponding tethering point with an initial conformation taken from single FG Nup simulations. In the case of ring and whole NPC simulations, each simulation was first energy-minimized in a three-step procedure to remove the possible clashes between the FG Nups. Then, each simulation was initiated with a 100 ns energy minimization run, followed by a 900 ns production run.

Langevin dynamics simulations were performed using Gromacs molecular dynamics simulation software v4.5.3 [40]. The cut-off distances for Van der Waals and Coulombic interactions are set to 2.5 nm and 5.0 nm, respectively. The cut-off length is defined as the maximum radial distance, in which the electrostatic and Van der Waals interactions are calculated for two beads. However, the effective electrostatic force is determined by the Debye screening parameter which is set to κ = 1.0 nm^-1^. The Langevin friction coefficient is set to 50 ps^−1^, similar to the collision frequency of water molecules [41]. Density plots of FG Nup rings were generated by assuming the ring is centered in a cylinder with a 100 nm diameter and a 10 nm length, which is discretized into 0.5x0.5x0.5 nm unit cells. In the case of the whole NPC, the cylinder has a diameter of 100nm and a length of 140nm. The number of occurrences of FG-repeats in each cell was counted over the total simulation time. Gridcount package was used to generate the density plots [42]. A time-step of 0.02 ps was used in simulations. All visualizations as well as end-to-end distance and root mean square deviation (RMSD) measurements were performed via VMD 1.9.1 [43].

## Results

### Like Charge Region (LCR) charged residues govern the size and dynamics of FG Nups

**Fig 1:**
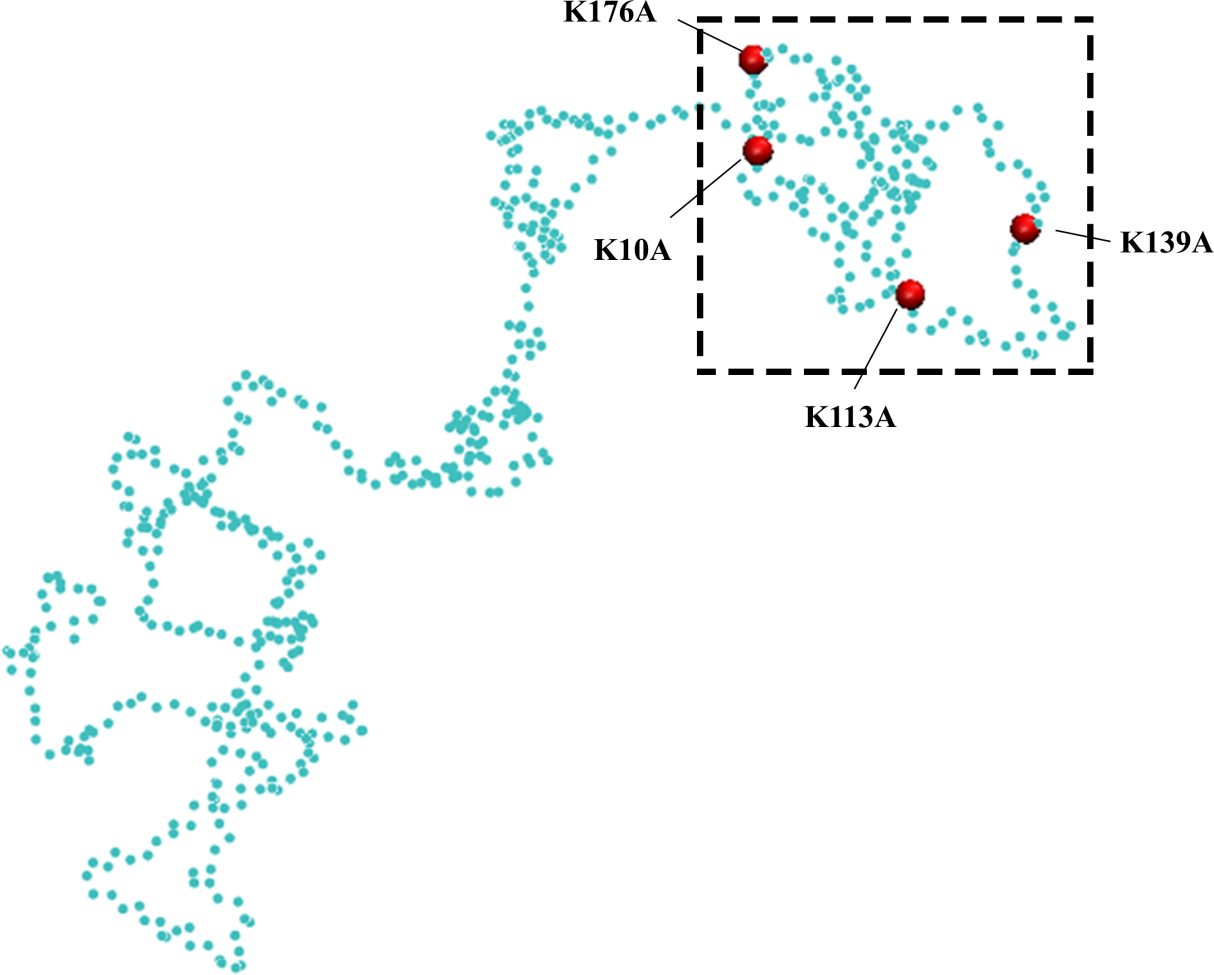
A random snapshot of Nsp1 simulation. The “like charge region” (LCR) (see text for definition) is boxed and demonstrates the four Lys residues that form the LCR of Nsp1. Mutated Nsp1 refers to the same sequence with these charged residues mutated to Ala.

**Fig 2:**
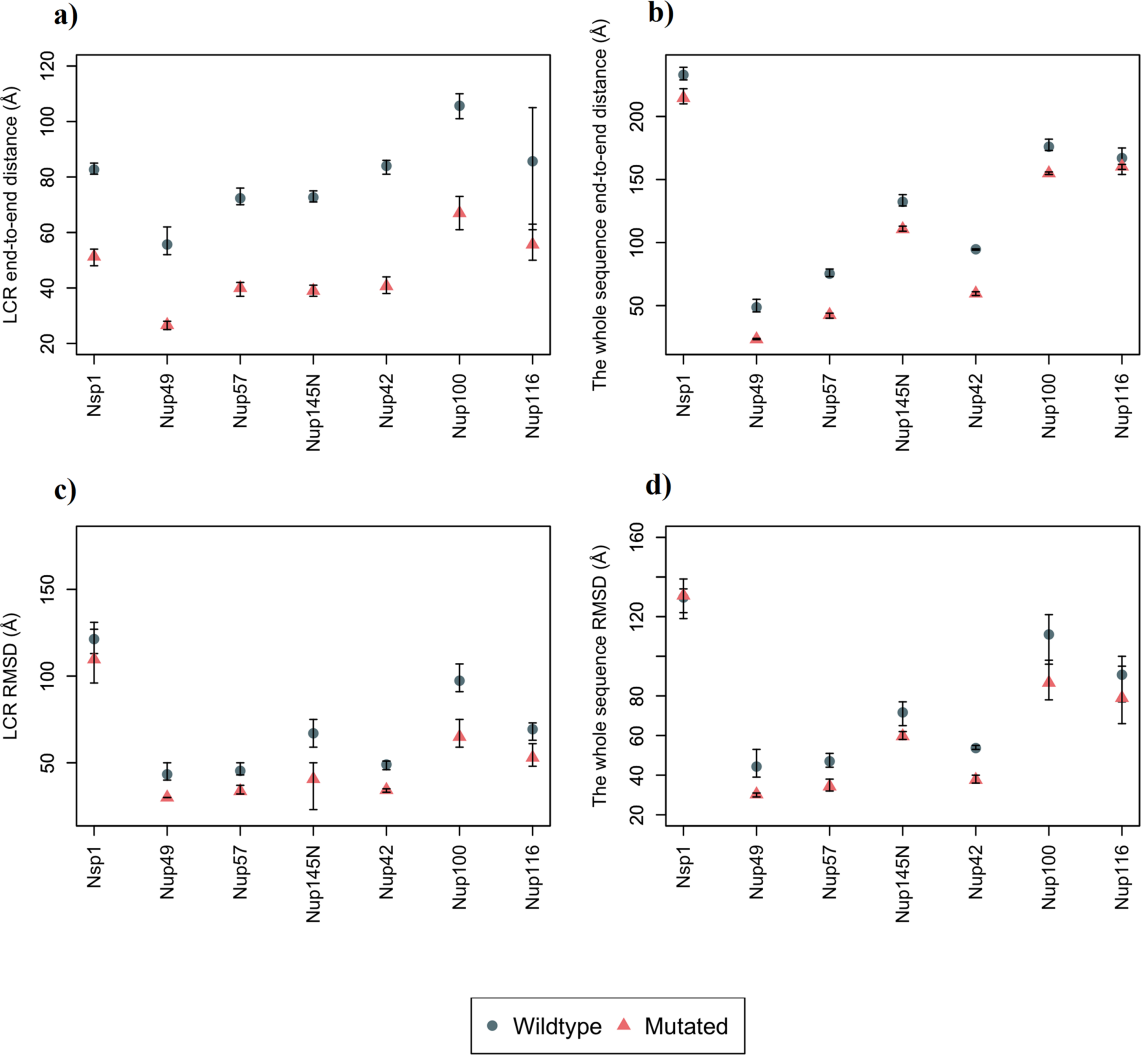
End-to-end distance and root mean square deviation (RMSD) measured for wildtype and mutated individual FG Nups. Mutated sequences represent a charged-to-Ala scanning within their like-charge regions (LCRs). Each simulation was repeated three times and the average and the error bars are represented in the graphs. (a) End-to-end distance of the LCR of FG Nups. Mutated sequences demonstrate a significant (35%-52%) reduction in end-to-end distance of their LCR compared to wildtype sequences, which demonstrates the role of LCR charges in maintaining the size of these regions. (b) End-to-end distance of the whole sequence of FG Nups. FG Nups with a long LCR compared to their length (Nup42, Nup49, Nup57) show nearly identical drop in end-to-end distance for LCRs as well as the whole sequence (37%-52%). On the other hand, FG Nups with a shorter LCR compared to their length, demonstrate a significantly lower drop for the end-to-end distance of the whole sequence (4%-17%). (c) RMSD of the LCR drops appreciably (9%-39%) in the mutated sequences, which suggests charges of LCRs as regulators of the highly dynamic nature of these regions. (d) RMSD for the whole sequence drops for up to 32% compared to the wildtype sequence.

We previously showed that the largest positively charged LCRs might play a regulatory role in FG Nup distribution[34]. As the first step, dynamics of yeast (*Saccharomyces Cerevisiae*) FG Nups, which contain positive LCRs (including Nsp1, Nup42, Nup49, Nup57, Nup100, Nup116, Nup145N) were individually explored in the present study. Hereafter, for the sake of simplicity, the largest positively charged LCRs would be referred to as LCRs. Each FG Nup was modeled both in wildtype and with its charged residues embedded within its LCR mutated to Ala (Fig 1). For the sake of accuracy, each simulation was repeated three times. Comparison of the physical behavior of these two configurations demonstrates the role of LCRs in FG Nup behavior. End-to-end distance as well as RMSD of the LCRs and the whole sequence were measured for each FG Nup (Fig 2), for the wildtype and mutated sequences. It is worth mentioning that LCRs of yeast FG Nups have a significant overlap with FG-rich regions[34].

Our results demonstrate that charges embedded in LCRs influence the size and conformational dynamics of these regions (Fig 2). The end-to-end distance of the LCR drops significantly upon the corresponding charged-to-Ala mutations. FG Nups with a long LCR compared to their length (Nup42, Nup49, Nup57) show nearly identical drop in end-to-end distance for the LCRs as well as the whole sequence. However, Nsp1, Nup100, Nup116, and Nup145N, which have relatively short LCRs with respect to the length of their entire sequence, exhibit a lower reduction in the end-to-end distance of the whole sequence compared to that of LCRs. This implies that the end-to-end distance of these Nups is primarily governed by the rest of the sequence, which means that regions containing LCRs maintain a more compact conformation as compared to the rest of the sequence. Our results are in agreement with previous studies, which reported that some of the yeast FG Nups, including Nsp1, Nup100, and Nup116, show two distinct domains with completely different physical characteristics [24,25]. One domain forms collapsed coil networks, while the other forms a stalk-like domain. In addition, variations in RMSD in LCRs as well as the whole sequences of mutated FG Nups demonstrate the influence of LCR charges on dynamics of FG Nups. Our previous study showed that LCRs overlap with FG-rich regions[34]. Therefore, here we conclude that LCRs of yeast FG Nups are the main regulators of size and dynamics of FG-rich regions.

### Analysis of FG Nup rings highlights the role of LCRs in FG Nup distribution

The NPC features an octagonal structure composed of spokes arranged in an eightfold rotational symmetric manner [44]. We divided the NPC into cross-sectional sections of different FG Nups to explore the mechanics of different regions (“rings”) of the NPC. Each ring consists of eight copies of a single FG Nup, arranged in an eightfold symmetric manner. Each ring was simulated for three times, each for 1μs, both in its wildtype as well as mutated forms. Since FG-repeats are suggested to be the main regulator of the active nucleocytoplasmic transport via weak interactions with cargo complexes [1,11,12], FG density plots were used as readouts of the simulations. Density of FG motifs was averaged over the simulation time for both wildtype and mutated rings to compare the two models. The results of the first trial of the simulations for Nup145N, Nup42 and Nsp1 are presented in the main text, while results of their second and third trial as well as simulations for the rest of the FG Nups, including Nup49, Nup57, Nup100 and Nup116, are presented in the supplementary material. It is worth mentioning that in these simulations, the blobs of the NPC scaffold are taken into account [28] which cause the empty space near the scaffold in some of the simulations.

#### Nup145N: LCRs promote cross-interactions between FG Nups

**Fig 3:**
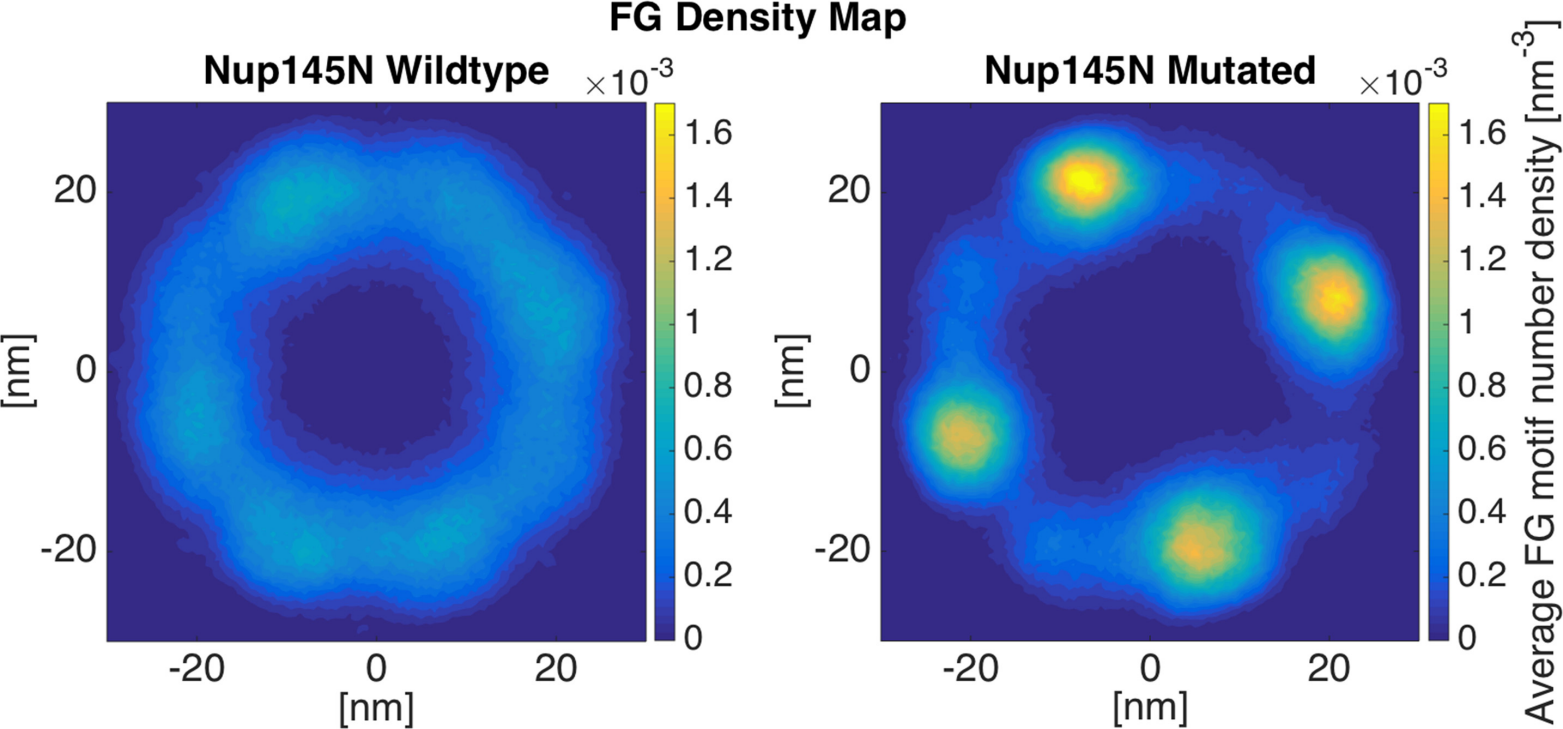
FG density plot of Nup145N ring. The wildtype ring shows a more even and inter-connected network of FG-repeats. On the other hand, FG network formed in the mutated ring is more aggregated to high-density regions with more limited interactions between different copies of Nup145N. This highlights the regulatory effect of like charge regions (LCRs) in effective distribution of FG-repeats inside the NPC. The rings are shown from the top view.

Nup145N is one of the relatively short FG Nups with a disordered region of 433-residues long. Its LCR (215-residues long) is half of the length of the whole sequence. According to Fig 2, mutation of LCR charges in Nup145N cuts its end-to-end distance of the LCR into half, while decreases the end-to-end distance of the whole molecule by just ~16%. Since LCRs are co-localized with FG regions[34], mutation of LCR charges in mutated ring leads to highly-dense FG regions (Fig 3). On the other hand, the wildtype ring exhibits a more even distribution of FG-repeats. The relatively short length of Nup145N causes its FG network to form a doughnut-like cloud, close to the NPC wall, rather than a network at the center [28]. Mutating charges in LCR not only causes the FG motifs to aggregate into high-density complexes, but also limits the interaction between different copies of Nup145N. The cross-interactions between Nup145N copies are limited and, consequently, they get trapped (in groups of two or three) into local minima of the energy landscape (S1 and S2 Videos). Since the repulsion between same charges does not exist anymore, the system does not get enough energy to jump out of these local minima and, hence, FG Nups remain interacting in the same groups to the end of the simulation. Reduction in interconnections between different copies of the FG Nup leads to restricted space covered by FG-repeats. These results are also discernible in the other two trials of the simulation, which confirms the regulatory role of LCRs (S1 Fig). As a result, one could postulate that the regulatory effect of LCRs in nucleocytoplasmic transport (in the case of Nup145N) is to increase the communication between different copies of the Nup and, subsequently, maximize the covered area by FG-repeats to facilitate the transport.

#### Nsp1: LCRs facilitate the formation of a larger FG-covered area

**Fig 4:**
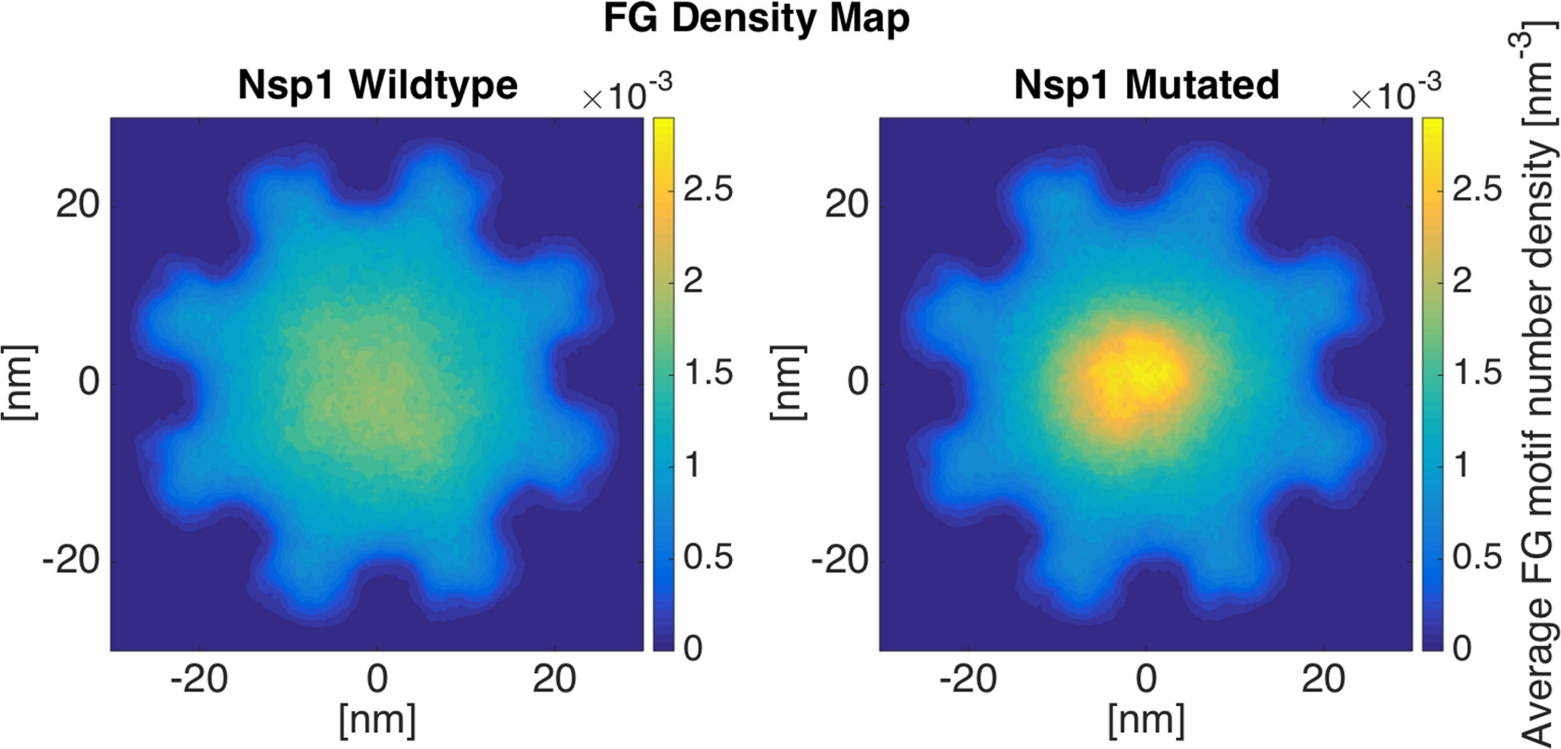
FG density plot of Nsp1 ring. FG density is distributed more evenly in the wildtype ring. A significant peak in FG density is observable in the mutant ring due to the collapse of the like charge region (LCR), which has a large overlap with the FG domain. Mutating charged residues in LCR allows the hydrophobic effect of the FG motifs to dominate, resulting in the formation of the high-density regions. These collapsed regions from all copies of Nsp1 stick together and form a relatively low-dynamic, high-density region. For the second and third trials of these simulations, please refer to S4 Fig.

Nsp1 is a relatively long FG Nup, containing 617 residues in its disordered region, while its LCR is only 167 residues long. According to the results from simulating individual wildtype and mutated Nsp1 sequences (Fig 2), mutation of LCR charges leads to a 39% decrease in the end-to-end distance of the LCR, while the end-to-end distance of the whole Nup does not change significantly. The same behavior is observable in the case of Nsp1 rings (Fig 4). The end-to-end distance of the Nsp1 copies does not change significantly; as the high-FG-density region is formed at the center in both cases [25,34] (wildtype and mutated). However, a considerable reduction in the end-to-end distance of the LCRs results in the formation of a relatively high-density region at the center of the NPC (Fig 4-Mutated). The wildtype ring shows a lower peak and a more even distribution of FG motifs compared to the mutated ring. The LCR mutated regions, which no longer contain charged residues, collapse into a more compact configuration and form a high-density region at the center of the ring, with the rest of the ring showing a relatively lower density of FG motifs. The reason underlying the formation of the density peak at the center of the ring is the long, highly-charged non-LCR region that forms an extended relaxed coil and brings the LCR (or in other words the FG domain) to the center of the NPC [25,34]. As the charges in the LCR are mutated, it collapses into a more compact form and allows the extension of the relaxed coil region (Fig 4-Mutated). The same behavior is observable in the case of Nup100 (S2 Fig) and Nup116 (S3 Fig). The other two trials of this simulation confirm these results as well S4 Fig.

#### Nup42: LCRs regulate the formation of FG cloud near the NPC wall

**Fig 5:**
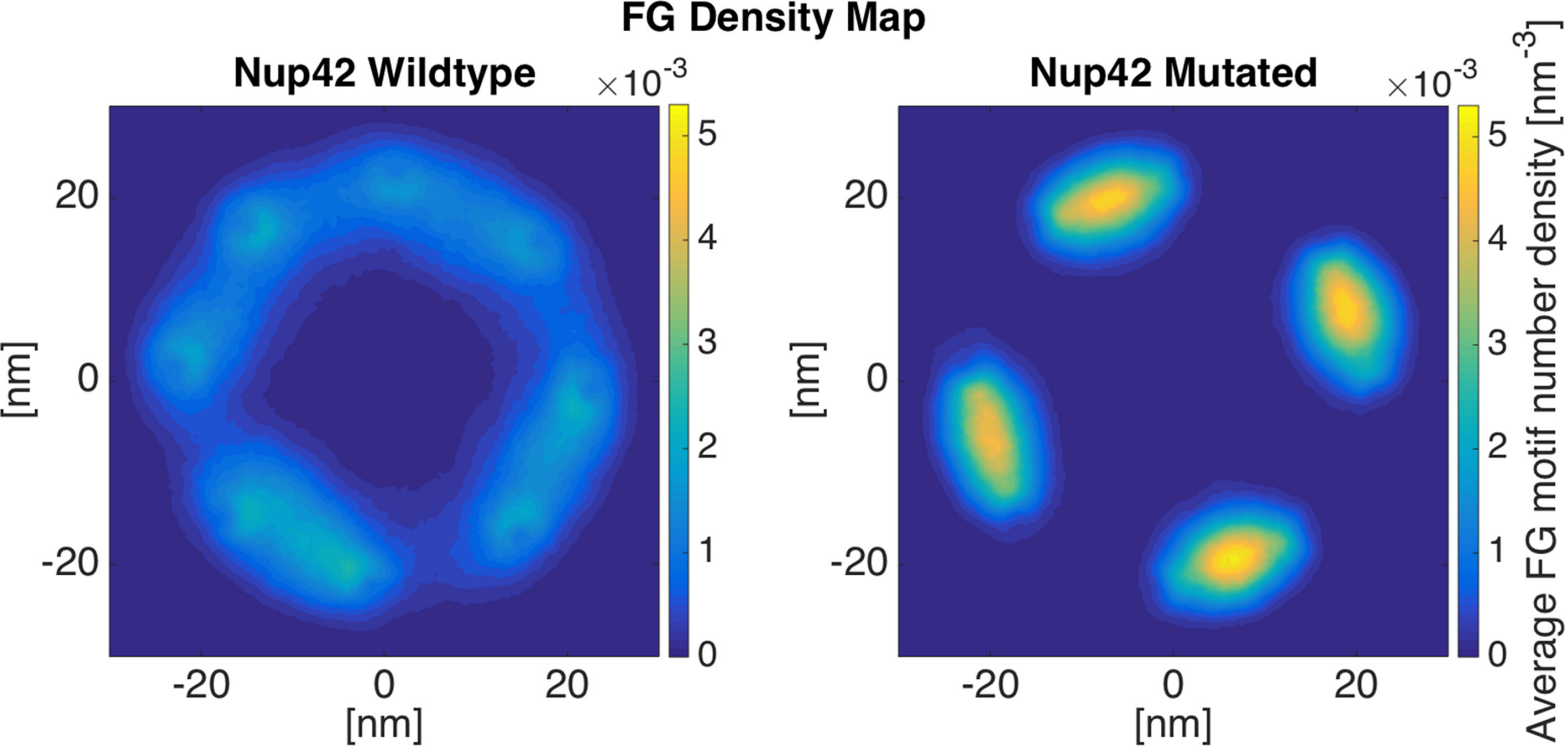
FG density plot of Nup42 ring. FG density shows a doughnut-like FG cloud that covers the near-the-wall space through dynamic inter-communication between different copies of Nup42. Mutated ring shows locally trapped groups of two Nups that are not able to escape the conditions due to the lack of dynamicity.

Nup42 is one of the relatively short FG Nups with a short disordered region, containing 382 residues. Nup42’s LCR is a 305-residue long domain, covering almost the whole disordered region of the Nup. Shorter Nups (Nup42, Nup49, and Nup57) mostly fluctuate near the wall of the NPC rather than covering the center of the pore (Fig 5, S5–S7 Figs). Therefore, the wildtype ring shows a doughnut-like high-FG density shape (Fig 5-Wildtype). Comparing the wildtype and mutated results, again, the lack of inter-communication between different copies of Nup42 in mutated ring results in local highly-dense regions, while leaving most of the space free of FG motifs. The same behavior is observed for Nup49 and Nup57 (S5 and S6 Figs). For the second and third trial of Nup42 simulation, please refer to S7 Fig.

To evaluate the functional role of LCRs and their regulatory effect in the behavior of FG Nups, a control simulation was designed. A new mutation was applied to the Nsp1 sequence, where four Lys residues (the same type and number of charges as in the LCR) were mutated to Ala in a non-LCR region of Nsp1. The mutated residues are located at positions 365, 369, 370 and 376. The results of this control simulation along with wildtype simulation are presented in S8 Fig. In contrast to the LCR mutation case, where the maximum density of FG repeats increased by 50% (as compared to that in the wildtype case) (Fig 4), the control mutation does not significantly change FG density distribution. The maximum density of FG repeats is altered by less than 10% compared to wildtype simulation. This further highlights the substantial regulatory role of LCRs in FG Nups distribution inside the NPC.

### The whole NPC: How LCRs assist FG Nups to dynamically cover the space inside the NPC

In order to see the overall effect of LCRs on the distribution of FG motifs inside the NPC, the whole NPC, including the hourglass-shape wall of the central channel and the disordered domains of all FG Nups was simulated using the coarse-grained molecular dynamics model. In the case of the mutant NPC, LCRs with positive charges were mutated in the corresponding FG Nups, i.e. Nsp1, Nup42, Nup49, Nup57, Nup100, Nup116, Nup145N. For the rest of the FG Nups (Nup1, Nup2, Nup159 and Nup60), which do not contain positive LCRs[34], wildtype sequences were used.

**Fig 6:**
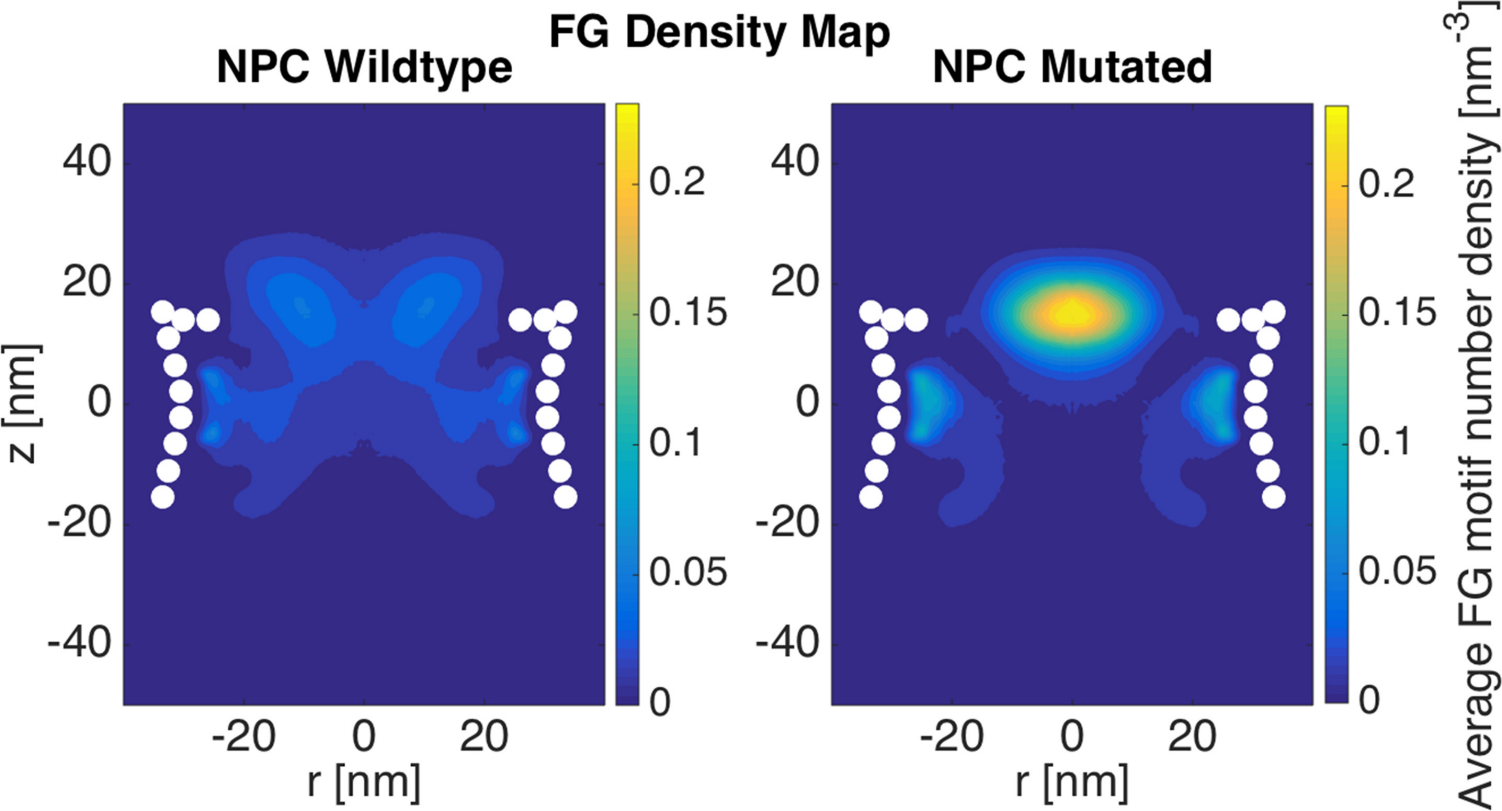
FG density plot of the whole NPC. Wildtype FG Nups form a doughnut-shaped high-density region at the entrance of the NPC[28], as well as a region next to the wall in the central channel with a slightly lower FG density. However, mutated like charge regions (LCRs) lead to a highly-dense, collapsed region at the center of the pore as well as near the wall in the central channel, while leaving the rest of the pore free of FG motifs. This comparison demonstrates the role of LCRs in promoting the dynamics of different FG Nups, resulting in a larger covered area.

Wildtype NPC shows a doughnut-shaped FG density plot, which was previously observed as well (Fig 6)[28]. The only difference is that the peak density is slightly shifted upward compared to the density plots in [28]. The reason is that while we used only the disordered regions of Nup49 and Nup57 [24] in our study, the whole sequences of these Nups were used in [28]. Therefore, it seems that using the whole sequences results in a smoother high-density region, where the longer sequences of Nup49 and Nup57 slightly pull down the high-density region that forms the doughnut-like region [28]. It was suggested that the doughnut-shaped FG distribution is compatible with the transportation mechanism observed by single molecule tracking experiments [45], where passive transport of small molecules occur through the center of the NPC. Despite modest differences, our results are in line with this proposed mechanism. In addition, the low-density central region of FG Nups is in line with experimental observation of a 10 nm empty hole at the center of the NPC [31]. Interestingly, mutation of charges in LCRs dramatically dysregulates this distribution (Fig 6). In the mutated NPC, the size of the high-density region is significantly shrunk and the peak of the density substantially increases. On the other hand, the rest of the space inside the NPC shows a very low density, implying that the dynamics of the system has significantly reduced and the system is almost frozen with very small vibrations. Therefore, the system has fallen into a system of collapsed FG regions and relatively straight stalk domains with significantly low dynamics. As a result, passive transport pathway is occluded by the high-density region and active transport pathway is disrupted due to the low dynamics of FG Nups and significantly smaller FG-covered area. It is noteworthy that all these observed dysregulations are due only to a handful of mutations (4–10) per sequence, demonstrating the regulatory role of LCRs.

## Discussion

### “LCR-regulated Coverage”

Through a bioinformatics study[34], we recently identified some evolutionarily conserved sequences of charged residues within the sequences of FG Nups that possess only positive charges, accordingly called “like charge regions” (LCRs). In this work, we sought to divulge physical significance of these specific domains of FG Nups in the distribution of FG-repeats inside the NPC and nucleocytoplasmic transport. We studied this role on yeast FG Nups and in three different levels, namely individual FG Nups, rings of FG Nups, and the whole NPC.

One of the most interesting aspects of the nucleocytoplasmic transport is the ability to handle more than a thousand cargos in every single second, and transport them with a dwell time of less than 10ms [46–48]. As a result, at every single snapshot, there are multiple cargos in the NPC and, consequently, in each ring of FG Nups. Therefore, each ring is required to accommodate transport of multiple cargos. Our results showed that wildtype rings exhibit a larger area covered by FG motifs, while mutated rings show a more collapsed FG network with the rest of the space left almost free (Figs 3–5). Considering that the NPC is a high throughput cellular machine, it should be able to accommodate multiple cargos at each time. Even distributions of FG repeats with a larger coverage inside the pore would facilitate more translocations. Wildtype rings are hence capable of translocating more cargos at each time step. Mutated rings represent a lower FG-covered area with some high FG density spots. This type of FG distribution might be more efficient for traffic of a few cargos per time step but is not necessarily effective for a high throughput selectivity offered by the NPC. This comparison focuses on transportation mechanism in a lower resolution, considering the transport requirements in each layer of the NPC in axial direction. Therefore, it could be speculated that LCRs are primarily co-localized with FG domains to maximize the dynamics of FG Nups and specifically regions of high FG density inside the NPC and facilitate a larger coverage of the central channel by FG-repeats. In other words, without LCRs, FG Nups would not be able to dynamically cover the whole space inside the pore (Fig 6) (since their FG domains collapse into compact forms), and cannot handle the numerous cargo-complexes passing through the NPC at each time step. This is in line with our individual FG Nup results as well, where mutation of charges in LCRs resulted in significantly lower RMSD and end-to-end distance (Fig 2).

**Fig 7:**
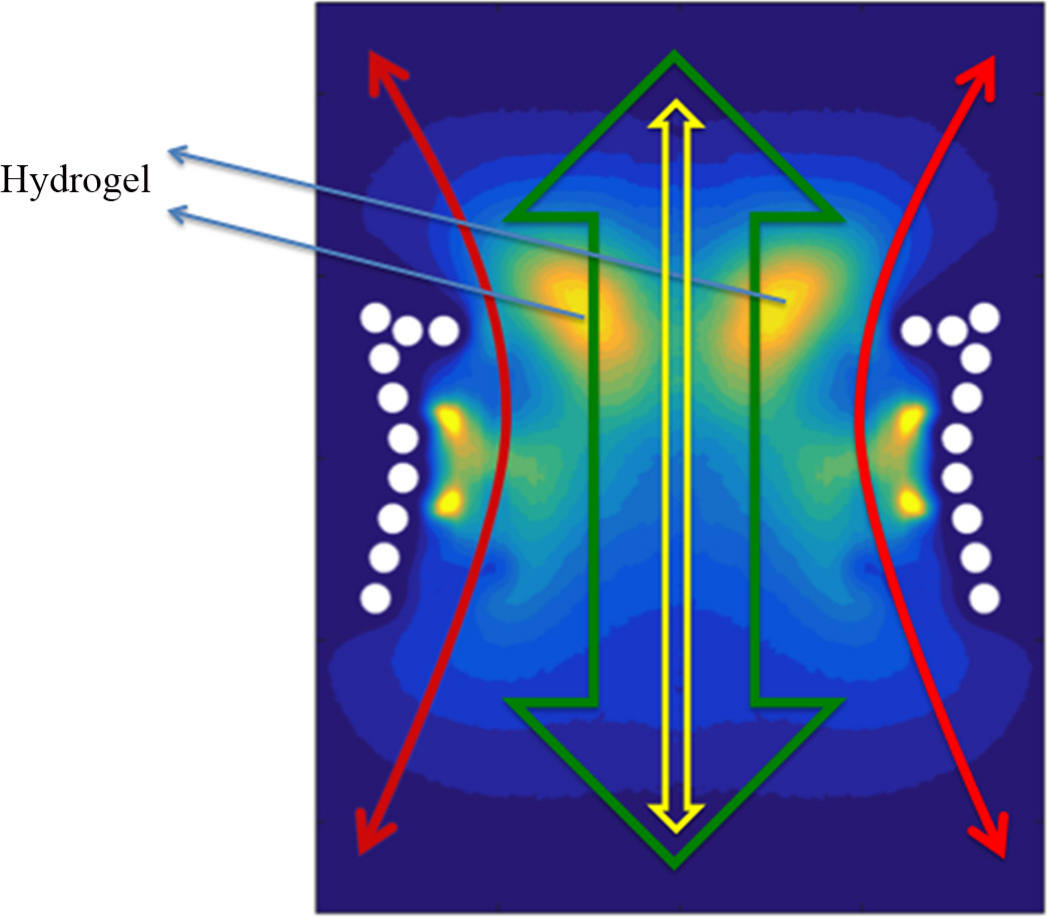
A schematic on top of the FG density plot of the wildtype NPC, showing different nucleocytoplasmic models observed in our results. According to the forest model [24], two transport pathways exist (green arrow for Zone1 and red arrows for Zone 2). Hydrogel model suggests that FG Nups form homogeneous FG-networks that transporters need to dissolve in order to pass through the NPC [19,20] (denoted by two blue arrows). Reduction-of-dimensionality, on the other hand, explains the transport as a 2D random-walk of transporters on an FG-rich surface over the wall of the central channel [23](red arrows). Our results demonstrated that like charge regions (LCRs) are the major regulator of the formation of these different regions for an efficient transport.

Our simulations demonstrated the categorization of FG Nups into two distinct groups[24,25,34]. While some of them (Nsp1, Nup100, and Nup116) bring their FG domain to the core of the central channel (Fig 4 and S2–S4 Figs), others (Nup42, Nup49, Nup57, and Nup145N) cover the area closer to the wall of the NPC (Figs 3 and 5, S1 and S5–S7 Figs). This observation is in line with the double-zone transport, previously suggested by Yamada et al. [24]. According to our model, Nsp1, Nup100, and Nup116 are among FG Nups that form a highly-dense FG region at Zone1 (Fig 7-green arrow) (leaving a low-density region at the center of the NPC (Fig 7-yellow arrow)), while others are responsible for transport through Zone2 (Fig 7-red arrows).

**Fig 8:**
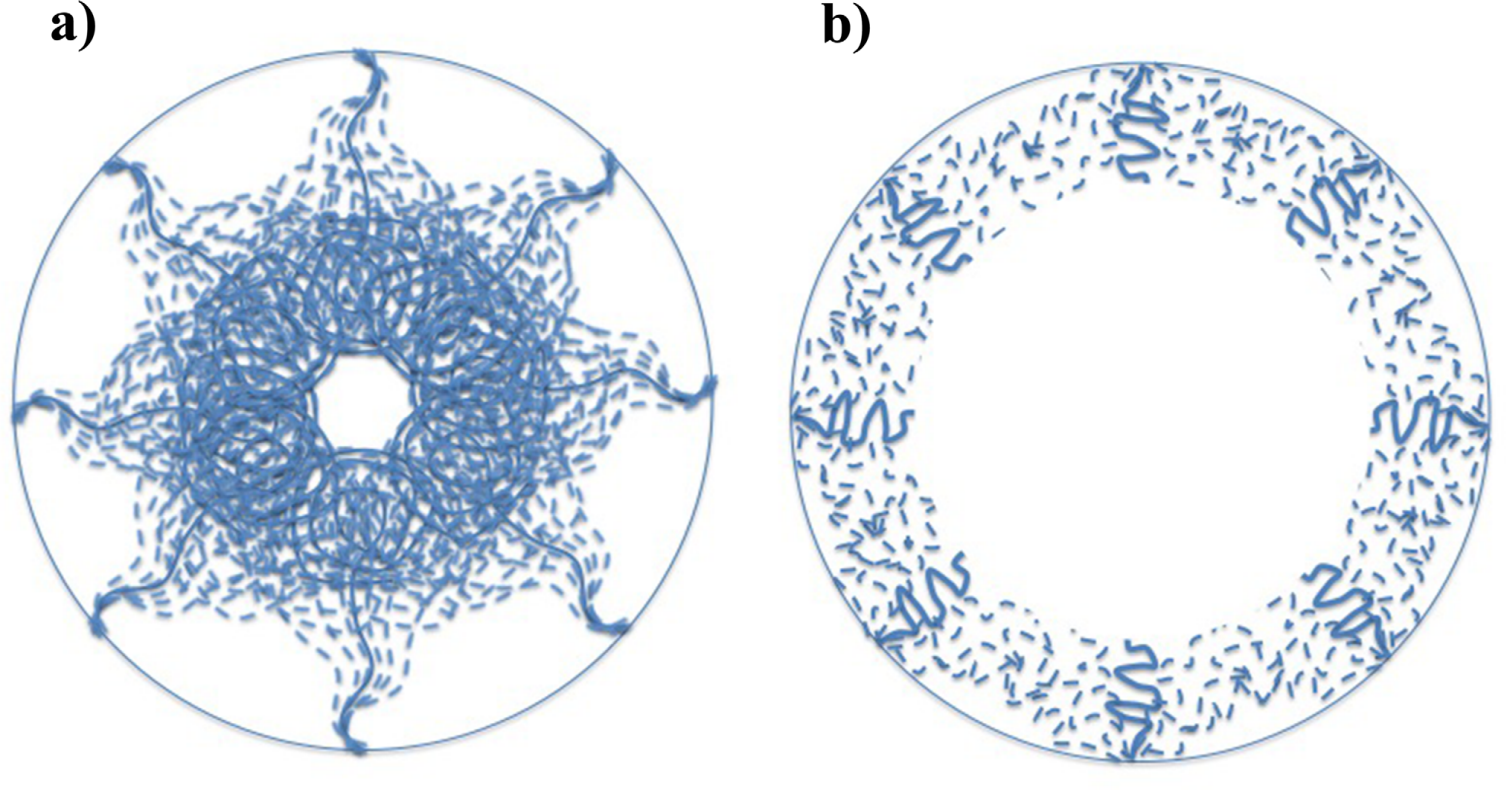
A schematic summarizing the role of LCRs in distribution of FG Nups inside the NPC, named “LCR-regulated Coverage”, LCR refers to like charge regions. Solid lines show a hypothetical initial configuration of FG Nups, while dotted lines show different configurations that FG Nups take over time. FG Nups are categorized into two groups. While one group (including Nsp1, Nup100, and Nup116 shown in (a)) bring their FG domain close to the center of the NPC [24,25] and collectively form a doughnut-like FG network, others (including Nup42, Nup49, Nup57, Nup145N shown in (b)) mostly cover the space next to the wall of the pore. In the first group, LCRs are primarily responsible for the size of the hydrogel formed in the middle (Fig 4), as well as a small opening at the very center of the NPC (Fig 6) [31]. On the other hand, in the second group, LCRs mainly promote interactions of FG Nups in the same layer to cover a larger space inside the pore (Figs 3 and 5).

On the other hand, our model supports the hydrogel model as well. The fact that rings of FG Nups form homogeneous meshwork inside the NPC (Figs 3–6), as opposed to sticking out without merging with each other [24], suggests that they may form a hydrogel (Fig 7-blue arrows) [32], where Kaps need to compete with FG-repeats to break FG-FG binds and travel through the NPC [32]. Our results did not specifically demonstrate the reduction-of-dimensionality model, however, this transport mechanism could be implied from our results. As seen in Figs 3 and 5 and 6, S5–S7 Figs, short FG Nups form an FG-rich region next to the wall of the central channel. It could be speculated that, since these regions are much smaller than the high-density region next to the center (Fig 6), rather than forming a hydrogel, they form a rich surface of nuclear transport receptors (NTRs) for transport of cargo-complexes. Therefore, rather than dissolving into this region, transporters slide on this surface. Collectively, one can postulate that the nucleocytoplasmic transport could be defined as a combination of three models, namely hydrogel, forest, and reduction-of-dimensionality models. According to the forest model, FG Nups are categorized into two groups, forming two distinct regions of active transport pathways (Fig 7-green and red arrows). Zone 1, which is closer to the center of the pore, is a doughnut-like structure with hydrogel characteristics for active transport (Fig 7-green arrow) and a low-density/empty space in between for passive transport [28,49] (Fig 7-yellow arrow). Zone 2, on the other hand, is an NTR-rich surface that enables a two-dimensional random walk for transporters (Fig 7-red arrows). In this work, we showed that formation of these different regions is highly dependent on LCRs to increase the dynamics of FG Nups for different purposes. While LCRs of some of the FG Nups, including Nsp1, mainly regulate the size of the hydrogel formed in the middle (Figs 4 and 6, S4 Fig), as well as a small opening at the very center of the NPC (Fig 6) [31,49], in other FG Nups, including Nup42, they facilitate interactions of different copies of FG Nups to cover a larger space inside the pore (Figs 3, 5 and 6). Accordingly, this model shall be referred to as the “LCR-regulated Coverage” (Fig 8), in which LCRs play a major role to enable FG Nups to form two potential pathways for cargo transport. However, further simulations including the cargos are required to lend more support to this model, which would be our future modeling study.

It is worth noting that structural and conformational characteristics of FG Nups are likely to be impacted by the presence of cargos. Nonetheless, the studies of the sequence, conformational dynamics, and biophysical behavior of FG Nups have been traditionally conducted in the absence of cargos[24–26,28,34]. Computational limitations have also hindered long-term simulation of these dynamic processes. Although 1μs is a considerable achievement for simulating the entire NPC, it may still be too short to capture a full picture of the FG Nup conformational dynamics. However, it should be noted that due to coarse-graining, time in our simulations is not representative of the physical time and should be multiplied by a speed-up factor to account for that [28]. Altogether, we hope that our study provides valuable insight about the mechanisms of nucleocytoplasmic transport and inspire further experimental and computational studies.

## Supporting Information

S1 Fig Three simulations of Nup 145N ring. Left figures represent wildtype rings and right figures represent LCR mutated rings. The wildtype ring shows a more even and inter-connected network of FG-repeats. On the other hand, FG network formed in the mutated ring is more aggregated to high-density regions with more limited interactions between different copies of Nup145N. The distribution of FG repeats is different in the mutated rings, which is due to the fact that in each simulation Nups are stuck in a different local energy minimum. However, in all sets of the simulations, the difference between wildtype and mutated rings represent the same regulatory role for LCRs.
(TIF)

S2 Fig Nup 100 is a relatively long sequence with a disordered domain of 800 residues with the LCR of 506 residues. This Nup behaves similar to Nsp1 and Nup116. The LCR mutation leads to a high FG density region in the center with a less FG covered area. Comparing the three trials of the simulation leads to the same conclusion about regulatory role of LCRs.
(TIF)

S3 Fig Nup 116 is similar to Nup 100 and Nsp1. It has a relatively long disorder region of 960 residues and contains a 571 residues long LCR. The LCR mutation leads to a high FG density region in the center with a less FG covered area. The three trials represent the same behavior.
(TIF)

S4 Fig Nsp1 rings. The first trial of the simulations is represented in Fig 4 in the main text. This figure shows the results of all the three trials together.
(TIF)

S5 Fig Nup 57 is a relatively short FG Nup, with 255 residues in its disordered region. Its LCR spans the whole disordered region. The wildtype ring shows that, due to the short length of the Nup, an FG-rich region is formed near the wall of the pore. However, in the mutated ring, the size of the FG-rich region considerably decreases, and FG density increases significantly. The three pairs of the figures are the results of the three trials for wildtype and mutated rings.
(TIF)

S6 Fig Nup49 has a 251-residues long disordered region, with a 199-residues long LCR. The same behavior as Nup57 is observable here. The three pairs of the figures are the results of the three trials for wildtype and mutated rings.
(TIF)

S7 Fig Nup42 rings. The first trial of the simulations is represented in Fig 5 in the main text. This figure shows the results of all the three trials together.
(TIF)

S8 Fig Control Simulation. A new mutation is applied to the Nsp1 sequence, where four Lys residues (the same type and number of charges as in the LCR) are mutated to Ala in a non-LCR region of Nsp1. The mutated residues are located at positions 365, 369, 370 and 376. In contrary to LCR mutation, where the maximum density of FG repeats increased by 50% (compared to wildtype), the control mutation does not significantly change FG density distribution. The maximum density of FG repeats is altered by less than 10% compared to the wildtype simulation. This further highlights the significant functional role of LCRs in FG Nup dynamics.
(TIF)

S1 Video Nup145N-ring-wildtype. (MPG)

S2 Video Nup145N-ring-mutated. (MPG)
