# Supplementary figures and images for: Nucleoporin's Like Charge Regions Are Major Regulators of FG Coverage and Dynamics Inside the Nuclear Pore Complex
Source: PLoS One. 2015 Dec 11;10(12):e0143745. doi: 10.1371/journal.pone.0143745 (PMC4676729; doi:10.1371/journal.pone.0143745)

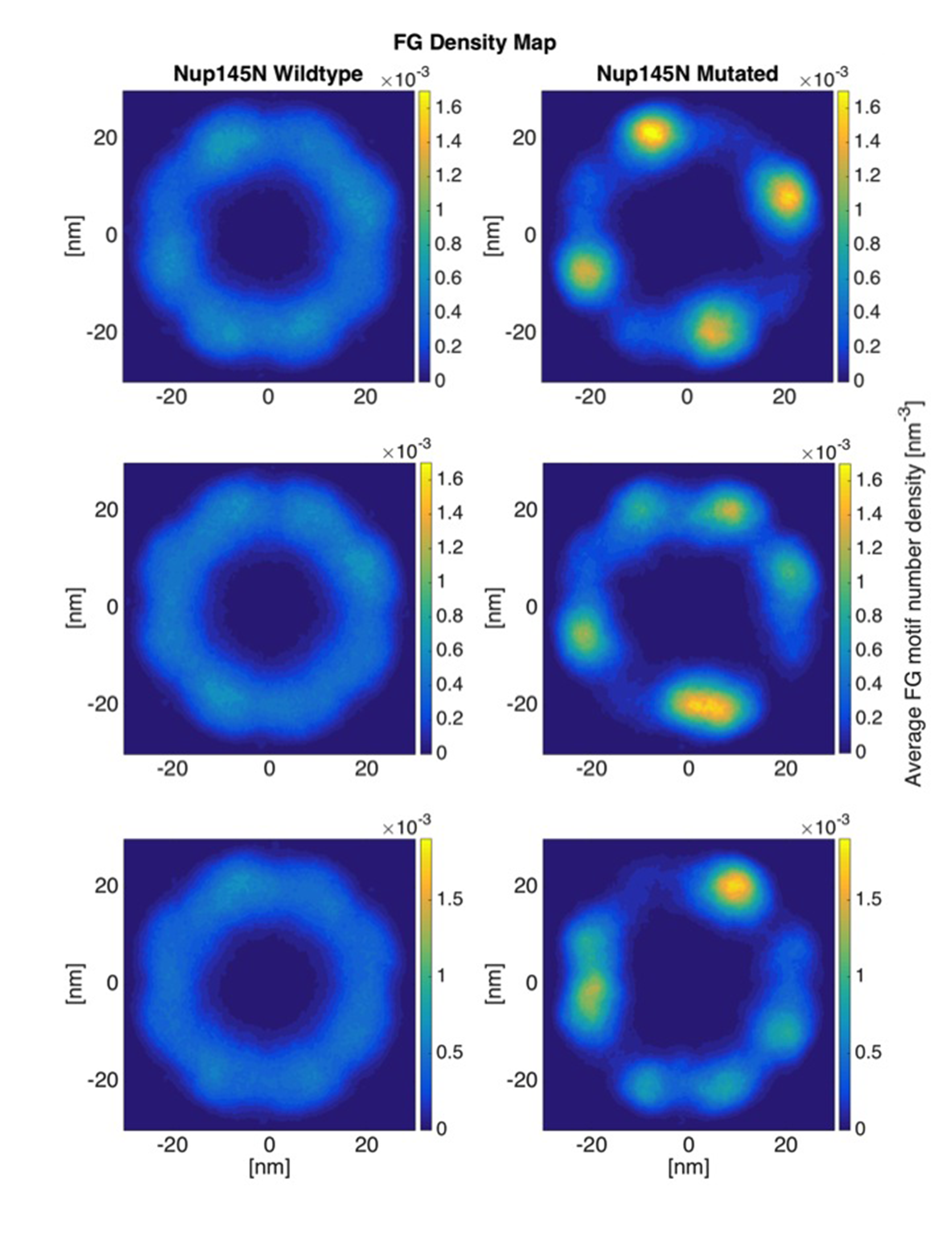

Supplement: S1 Fig — Left figures represent wildtype rings and right figures represent LCR mutated rings. The wildtype ring shows a more even and inter-connected network of FG-repeats. On the other hand, FG network formed in the mutated ring is more aggregated to high-density regions with more limited interactions between different copies of Nup145N. The distribution of FG repeats is different in the mutated rings, which is due to the fact that in each simulation Nups are stuck in a different local energy minimum. However, in all sets of the simulations, the difference between wildtype and mutated rings represent the same regulatory role for LCRs. (TIF) [file pone.0143745.s001.tif]

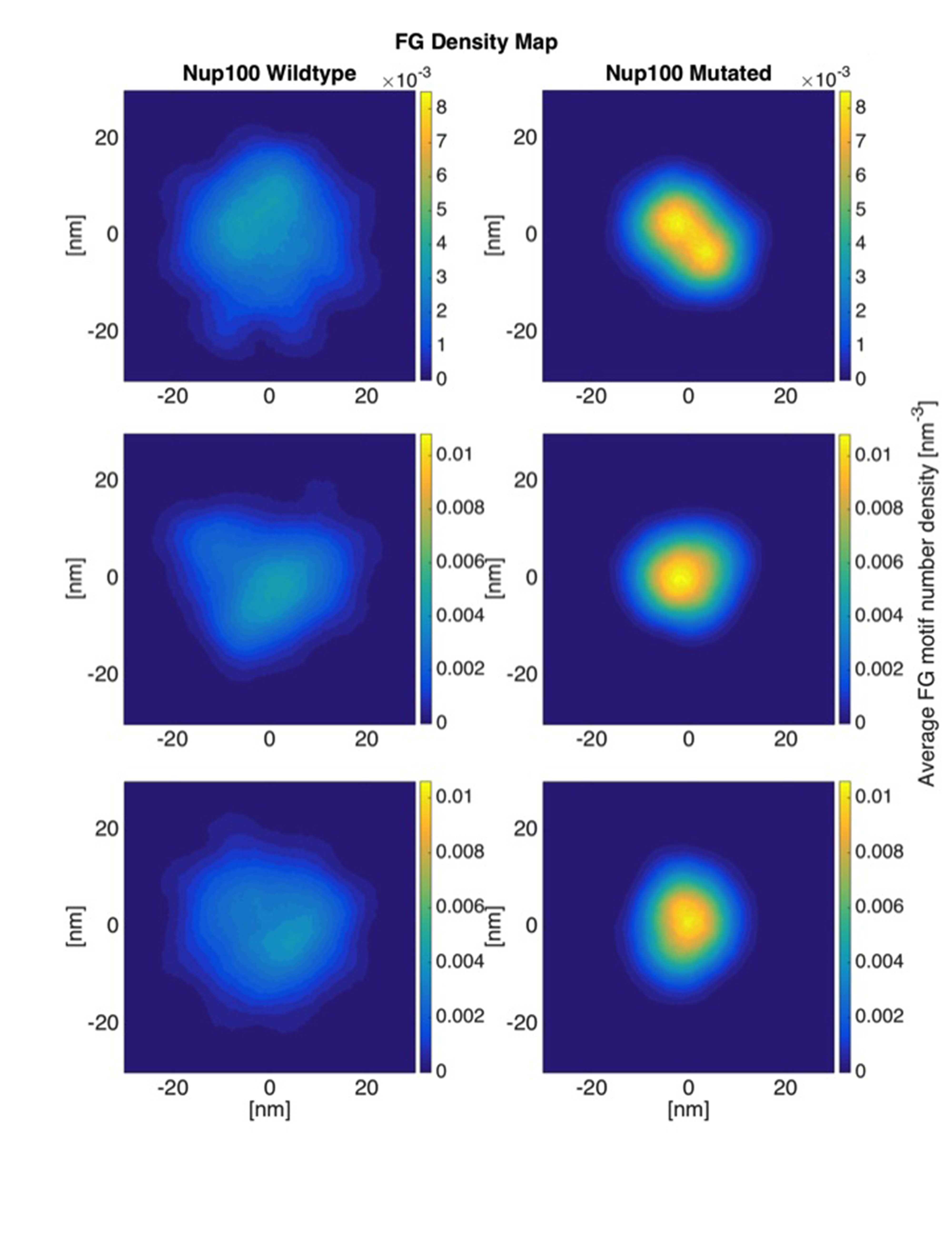

Supplement: S2 Fig — This Nup behaves similar to Nsp1 and Nup116. The LCR mutation leads to a high FG density region in the center with a less FG covered area. Comparing the three trials of the simulation leads to the same conclusion about regulatory role of LCRs. (TIF) [file pone.0143745.s002.tif]

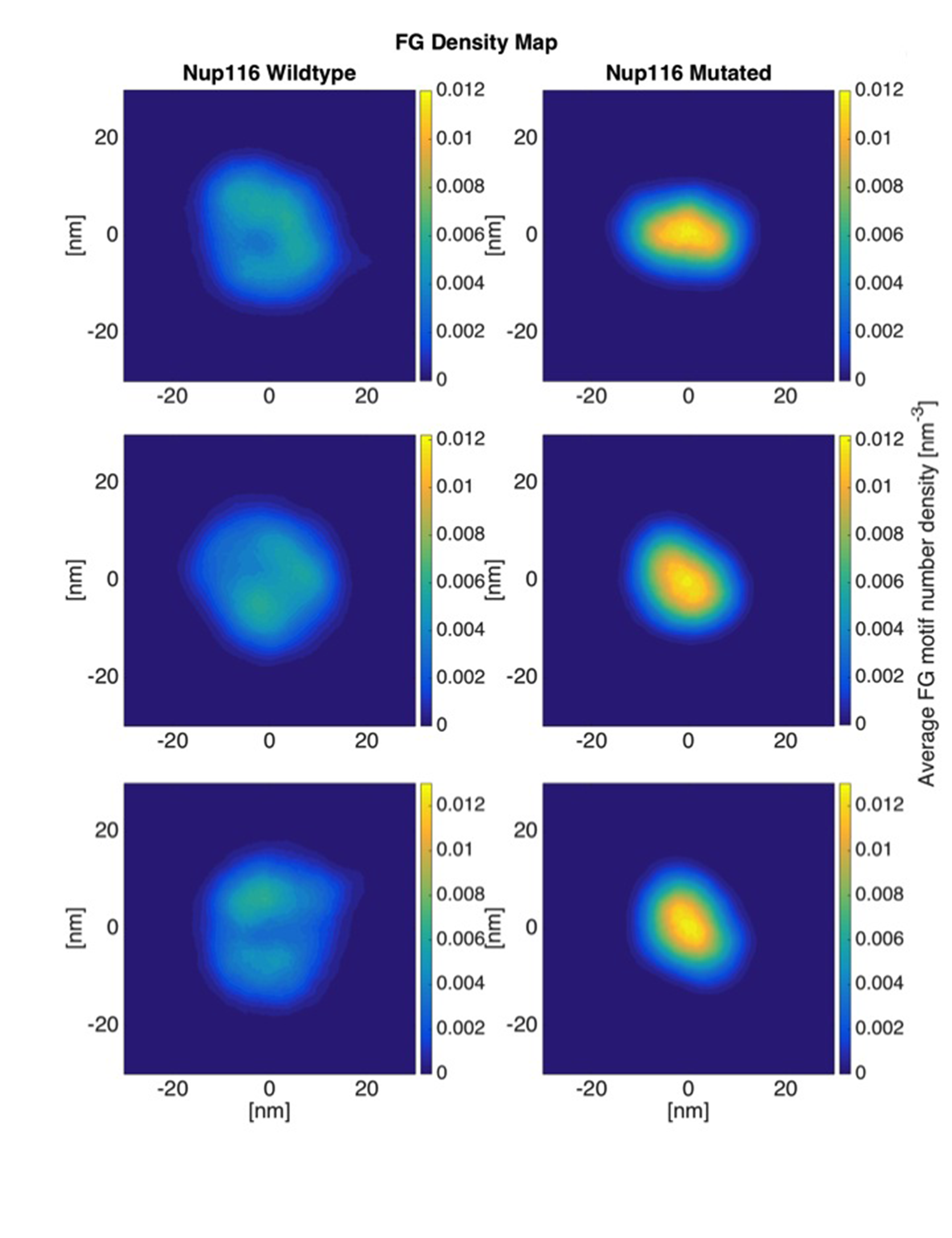

Supplement: S3 Fig — It has a relatively long disorder region of 960 residues and contains a 571 residues long LCR. The LCR mutation leads to a high FG density region in the center with a less FG covered area. The three trials represent the same behavior. (TIF) [file pone.0143745.s003.tif]

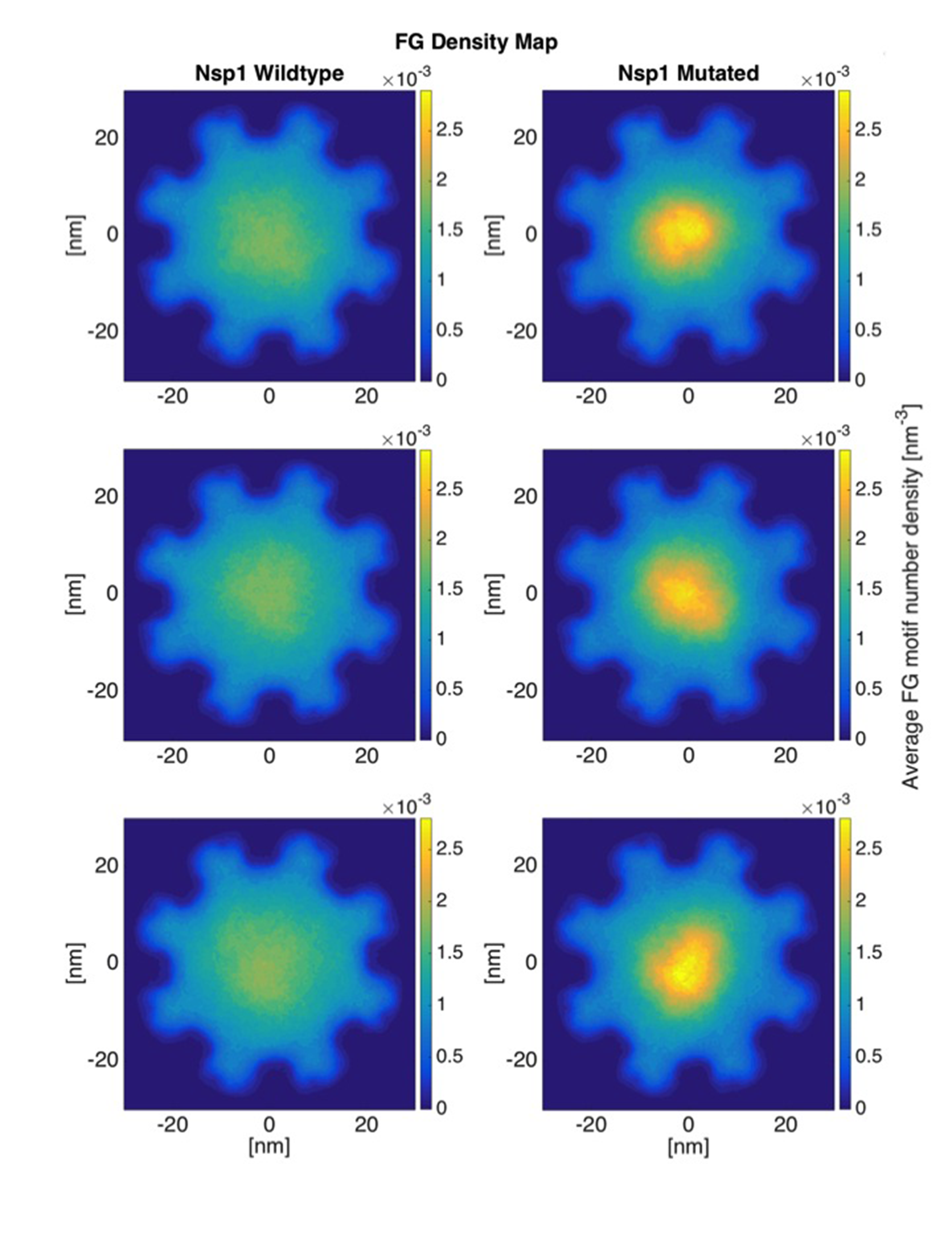

Supplement: S4 Fig — The first trial of the simulations is represented in Fig 4 in the main text. This figure shows the results of all the three trials together. (TIF) [file pone.0143745.s004.tif]

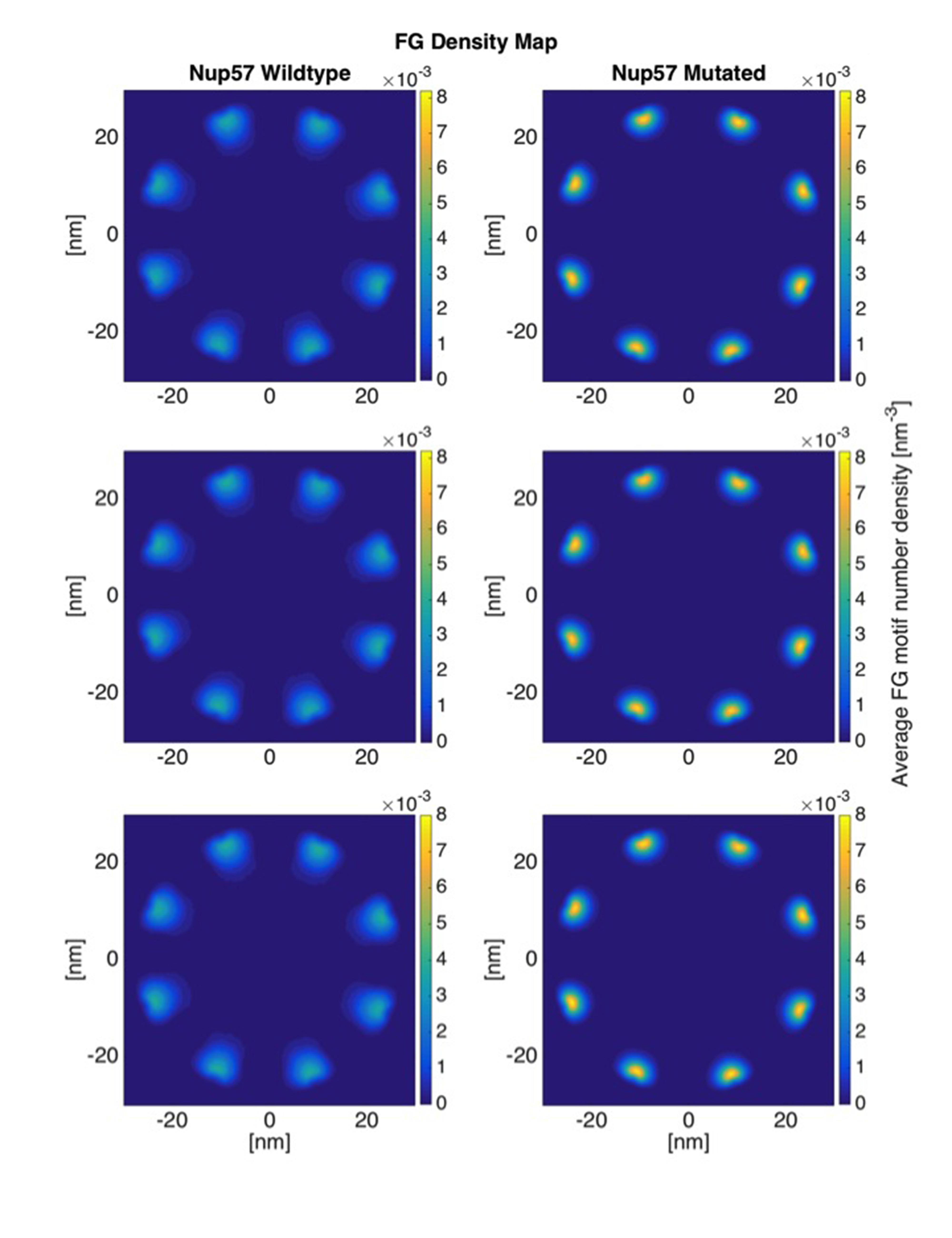

Supplement: S5 Fig — Its LCR spans the whole disordered region. The wildtype ring shows that, due to the short length of the Nup, an FG-rich region is formed near the wall of the pore. However, in the mutated ring, the size of the FG-rich region considerably decreases, and FG density increases significantly. The three pairs of the figures are the results of the three trials for wildtype and mutated rings. (TIF) [file pone.0143745.s005.tif]

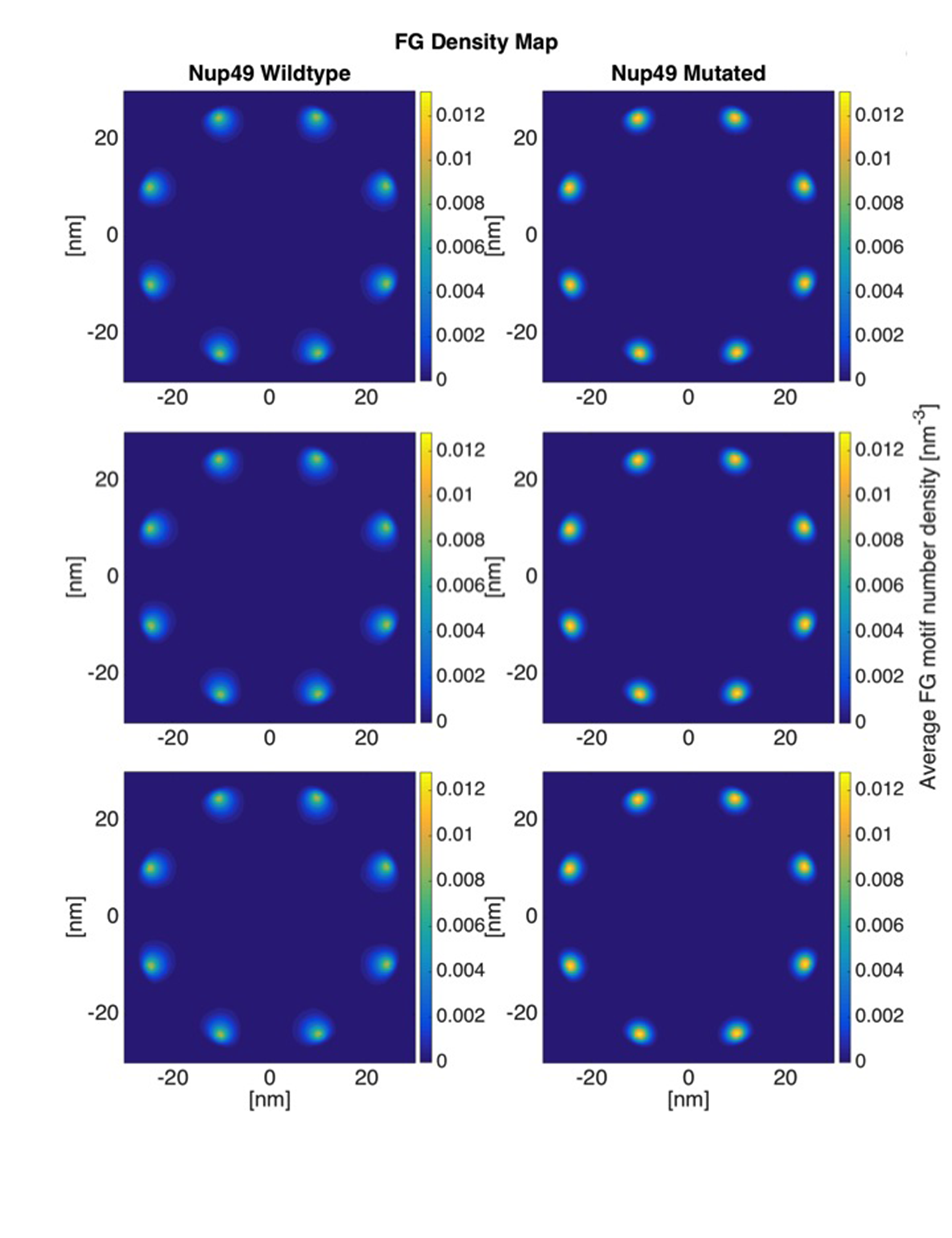

Supplement: S6 Fig — The same behavior as Nup57 is observable here. The three pairs of the figures are the results of the three trials for wildtype and mutated rings. (TIF) [file pone.0143745.s006.tif]

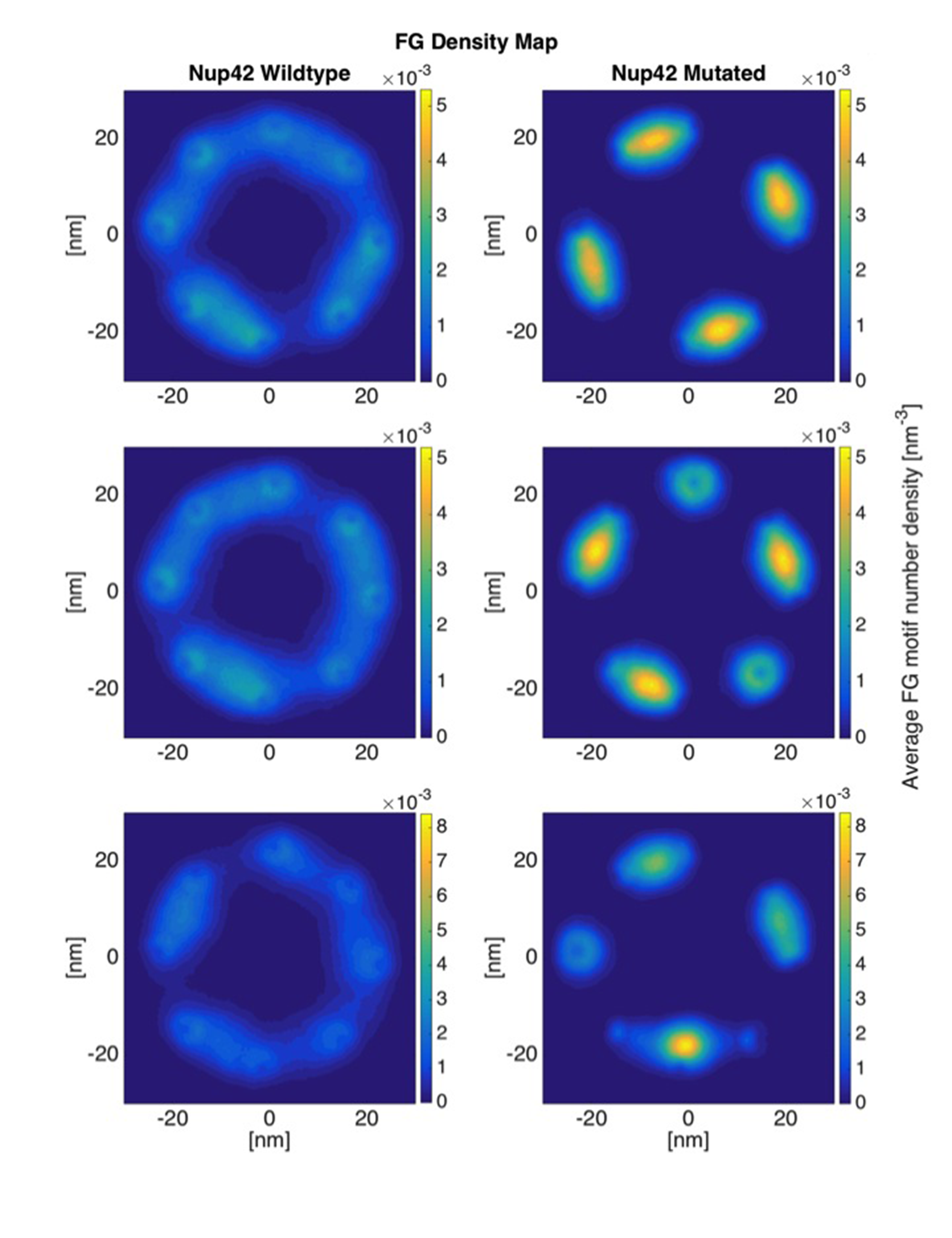

Supplement: S7 Fig — The first trial of the simulations is represented in Fig 5 in the main text. This figure shows the results of all the three trials together. (TIF) [file pone.0143745.s007.tif]
